# Preliminary Study of Prospective ECG-Gated 320-Detector CT Coronary Angiography in Patients with Ventricular Premature Beats

**DOI:** 10.1371/journal.pone.0038430

**Published:** 2012-06-06

**Authors:** Tong Zhang, Jinquan Bai, Wei Wang, Dan Wang, Baozhong Shen

**Affiliations:** 1 Department of Radiology, The Fourth Affiliated Hospital, Harbin Medical University, Harbin, China; 2 Department of MRI Room, The First Affiliated Hospital, Harbin Medical University, Harbin, China; University Hospital of Würzburg, Germany

## Abstract

**Background:**

To study the applicability of prospective ECG-gated 320-detector CT coronary angiography (CTCA) in patients with ventricular premature beats(VPB), and determine the scanning mode that best maximizes image quality and reduces radiation dose. **Methods:** 110 patients were divided into a VPB group (60 cases) and a control group (50 cases) using CTCA.All the patients then underwent coronary angiography (CAG) within one month. CAG served as a reference standard through which the sensitivity, specificity, positive predictive value (PPV), and negative predictive value (NPV) of CTCA in diagnosing significant coronary artery stenosis (luminal stenosis ≥50%) could be analyzed. The two radiologists with more than 3 years’ experience in cardiac CT each finished the image analysis after consultation. A personalized scanning mode was adopted to compare image quality and radiation dose between the two groups.

**Methodology/Principal Findings:**

At the coronary artery segment level, sensitivity, specificity, PPV, and NPV in the premature beat group were 92.55%, 98.21%, 88.51%, and 98.72% respectively. In the control group these values were found to be 95.79%, 98.42%, 90.11%, and 99.28% respectively. Between the two groups, specificity, sensitivity PPV, NPV was no significant difference. The two groups had no significant difference in image quality score (P>0.05). Heart rate (77.20±12.07 bpm) and radiation dose (14.62±1.37 mSv) in the premature beat group were higher than heart rate (58.72±4.73 bpm) and radiation dose (3.08±2.35 mSv) in the control group. In theVPB group, the radiation dose (34.55±7.12 mSv) for S-field scanning was significantly higher than the radiation dose (15.10±1.12 mSv) for M-field scanning.

**Conclusions/Significance:**

With prospective ECG-gated scanning for VPB, the diagnostic accuracy of coronary artery stenosis is very high. Scanning field adjustment can reduce radiation dose while maintaining good image quality. For patients with slow heart rates and good rhythm, there was no statistically significant difference in image quality.

## Introduction

Coronary CTA is currently the first choice for non-invasive diagnosis of coronary artery disease (CAD). This method has high diagnostic accuracy [1], [2], but its accuracy is influenced by heart rate, rhythm, and other factors. The width coverage of 320-detector CT is 16cm, so a single CT snapshot can cover the whole heart. By avoiding overlapping scanning, the radiation dose received by the patient can be reduced. Additionally, 320-detector CT shortens acquisition time. Because scanning can be completed shortly after bolus injection of a contrast agent, the amount of contrast agent used can also be reduced. Although the time resolution of 320-detector CT is not higher than that of dual-source CT, the former can overcome this shortcoming through the use of multi-sector reconstruction, and with two-sector acquisition time resolution can reach 87.5 ms [3]. Currently, prospective ECG-gated 320-detector CTCA has very high diagnostic accuracy for subjects with heart rates below 65 bpm, and radiation dose is also low[4]–[6]. Because the technique works best at lower heart rates, patients with heart rates above 65 bpm can be given an oral-blockade (50–100 mg metoprolol) one hour before a CT examination. Arrhythmia, however, poses additional challenges to maximizing image quality while reducing radiation dose. Some reports have shown that prospective ECG-gated CTCA is inappropriate for 64-detector CT and dual-source CT when scanning subjects with heart rates above 70 bpm or significant heart rate change (e.g. arrhythmia) [7], [8]. In clinical settings, ventricular premature beat is very common. Whether physiological or pathological in nature, it negatively impacts scanning results. If premature beats occur when scanning, it disrupts the prospective scheduled scanning program and will result in more severe motion artifacts. However, if we pause scanning during the premature phase and acquire data during the next normal phase or additionally scan the next R-R interval, then we can achieve fair results. In this study, prospective ECG-gated 320-detector CT is adopted to scan patients with ventricular premature beats. Patients with heart rates below 65 bpm were placed in a control group. Both groups underwent coronary angiography to evaluate the diagnostic accuracy of 320-detector CTCA for significant (≥50%) stenosis and access the technique’s ability to maintain good image quality while reducing radiation dose.

## Materials and Methods

### Objects

This study was approved by the Ethics Committee of Harbin Medical University. Data from 110 patients undergoing 320-detector CT coronary angiography in our hospital from October 2009 to June 2011 were collected (72 males and 38 females, aged 38- to 86-years-old, mean age 59-years-old). These patients were divided into two groups, those with ventricular premature beats (60 cases) and those with a normal rhythm and a heart rate below 65 bpm (50 cases). The criterion of patients with ventricular premature beat is that the acquisition range was just in time to catch ventricular premature beat during prospective ECG-gated scanning. 50 cases were detected during medium-field (M-field, FOV = 320 mm) scanning, and 10 cases were detected during small-field (S-field,FOV = 200 mm) scanning. Patients in the later group were found to have a normal rhythm and a heart rate below 65 bpm through M-field scanning. All the patients underwent CAG within one month. The exclusion criteria for both groups were that patients (1) were unable to complete the breath-hold action, (2) had a heart rate above 65 bpm and a normal rhythm, (3) suffered from arrhythmias other than ventricular premature beat, (4) were allergic to iodine contrast agents, (5) had severe renal insufficiency, and (6) had decompensated cardiac insufficiency. All patients with heart rates above 65 bpm were administered Betaloc (50–100 mg) orally. The detailed basic parameters of the subjects are shown in Table 1. This study was approved by the local Ethics Committee and informed consent forms were signed by all patients.

**Table 1:** Patient characteristics.

Characteristics	
Age (years old)	59±9 (38–86)
Male/female	72/38 (total 110)
BMI (kg/m2)	25.39±2.81 (20.5–34.5)
Hypertension (120/80 mmHg)	68/110 (62%)
Hyperlipaemia (>200 mg/dl)	41/110 (37%)
Obesity*	35/110 (32%)
Smoking history	45/110 (41%)
Family history of coronary heart disease	31/110 (28%)
Diabetes mellitus	21/110 (19%)
After stenting	19/110 (17%)
After bypass	8/110 (7%)

Variables are expressed with frequency ± standard deviation, and others are expressed with percentage.

* BMI(Body mass index)≥30 kg/m^2^ is obesity.

### CTA and Scanning Parameters

A Toshiba Aquilion One 320-detector CT scanner was used, with detector collimation at 320×0.5 mm and coverage in Z direction set at 16 cm. A prospective ECG-gated technique and arrhythmia rejection algorithm (Toshiba Medical Systems, Nasu, Japan) were adopted. Patients with no allergic reaction to the contrast agent were placed on the scanning bed, connected to the ECG monitor, positioned, and given breathing training. A normal saline line was administered and checked to ensure that the pressure was suitable and the venous catheter was unblocked. The ECG leads (standard II ECG leads were used) were adjusted to optimize the distinction between R waves and other waves, making the R wave, which triggers prospective ECG gating, easily identifiable, at the same time observing in electrocardiogram whether ventricular premature beat appears. Scanning field of view(FOV) was selected according to the size of subjects’ hearts. Available scanning FOV included medium field (M-Field, FOV = 320 mm), and small field (S-field, FOV = 200 mm). The range was generally from the trachea carina to the heart bottom, covering the whole heart. Manual trigger scan mode was adopted, with the monitoring level at the midline level in the scanning field. Each patient’s ECG was observed during scanning to check whether premature beats occurred. Premature beats were avoided during scanning so that image quality was preserved. After injecting the contrast agent, the scanning began when the left ventricle was light and the right ventricle was dark. The relevant parameters were as follows: different tube voltage and tube current was set according to patient body mass index (BMI) [9] (Table 2). A non-ionic contrast agent, iobitridol, was administered in a (370 mgI/ml) 40–60 mL dose. The flow rate was generally 6.0 mL/s, and after injection a flush with 30 mL of normal saline was performed. Prospective gating CTCA of all the patients with heart rates <65 bpm were performed from 70% to 80% of the R-R interval. The acquisition range for patients with ventricular premature beats and heart rates ≥65 bpm were expanded to 30%–80%, and the best time phase was then selected for evaluation.

**Table 2:** Tube voltage/tube current adjustment table based on BMI.

100kv		120kv		135kv	
BMI	mA	BMI	mA	BMI	mA
17	450	23	400	40+	510
19	500	25	450		
21	520	30	500		
23	550	35–40	580		

BMI = mass(kg)/[height(m)]^2^, unit: kg/m^2^.

### Data Processing

After scanning, the machine automatically generated a volume image with a relative time phase of 75% and automatically reconstructed a volume image of the absolute time phase according to the collected data. If the images were not good, ECG would be used to edit and select the period phase with a longer and fixed R-R interval for reconstruction. Shorter or non-fixed period phases would be removed [10]. A Vitreal FX post-processing workstation was used to restructure and analyze the coronary artery. Restructuring modes included volume render (VR), curved planar reformation (CPR), and maximum intensity projection (MIP). Through the software’s automatic measuring function, the degree of coronary artery stenosis was determined through the following calculation: (%)  =  [(inner diameter of normal vessel proximal to stenosis segment + inner diameter of normal vessel distal to stenosis segment)/2– inner diameter at the narrowest position of stenosis segment]/[(inner diameter of normal vessel proximal to stenosis segment + inner diameter of normal vessel distal to stenosis segment)/2] × 100%. Segments with an obtained stenosis degree ≥50% were compared and analyzed with CAG.

### Image Evaluation and Analysis Method

**Figure 1:**
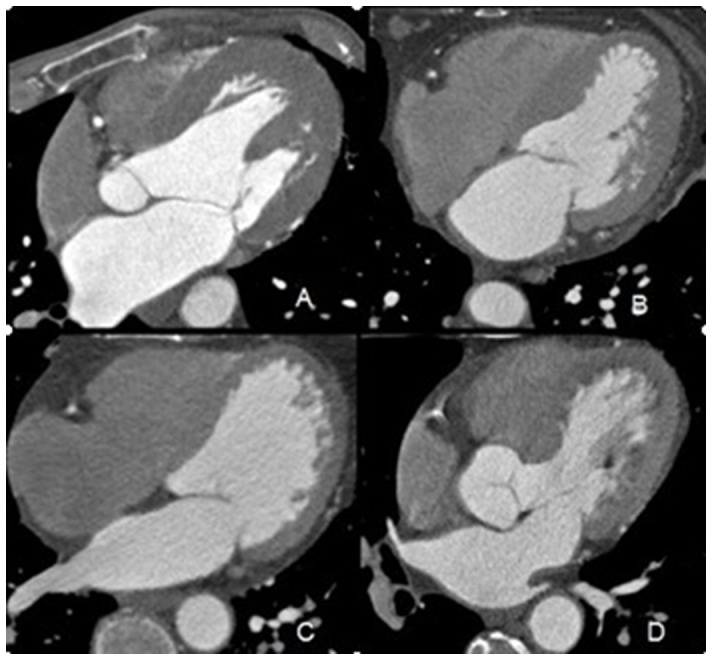
Axial A-D of middle segment of the RCA are respectively scored as 4–1: 4, excellent, no artifacts; 3, good, mild artifacts; 2, acceptable, moderate artifacts, but can still make a diagnosis; and 1, cannot evaluate, severe artifacts, cannot make a diagnosis.

According to the classification criteria issued by the American Heart Association (AHA), the coronary artery is divided into 15 segments [11]. CTCA image quality is divided into four grades: 4, excellent, no artifacts; 3, good, mild artifacts; 2, acceptable, moderate artifacts, but a diagnosis is still possible; and 1, cannot evaluate, severe artifacts, a diagnosis is not possible (Figure 1). All 15 segments of the coronary artery with a luminal diameter ≥1.5 mm were scored. Two reviewers (with more than 3 years’ experience in cardiac CT each) who were blinded to the CT protocol and the results of CCA independently assessed the reconstructed images. After their separate reading sessions, the discrepancy was resolved during a third session, in which the reviewers read images together at the same time to reach a consensus.

### CAG

A Philips FD 20 digital flat-panel angiography machine and 5–6F Guiding Catheter were used. After injecting the non-ionic iodinated contrast agent, left coronary angiography with four projection positions and right coronary angiography with two projection positions were conducted. The obtained images were stored, and measuring software was used to calculate the degree of coronary artery stenosis. The method of calculation was the same as used in CTCA image processing.

### Statistical Analysis

SPSS 17.0 statistical software was used to analyze the collected data. Age, heart rate, radiation dose, and other variables were expressed with mean and standard deviation, and an independent sample t test was used for comparison. With CAG as the reference standard, sensitivity, specificity, PPV, and NPV of CTCA stenosis were evaluated respectively at both the patient and segment levels (every segment of every vessel). Then the Pearson chi-square analysis were used to analyze the statistical differences of specificity, sensitivity, PPV, NPV between the two groups. Pearson correlation coefficients were used to analyze the correlation between heart rate and radiation dose in the premature beat and control groups. All analyses use a two-sided test, and *P*<0.05 were set as a threshold for statistical significance.

## Results

### CTCA Accuracy

At the patient level, sensitivity, specificity, PPV, and NPV in the premature beat group were 100%, 94.59%, 86.66%, and 100% respectively. Analogous values in the control group were found to be 100%, 95.24%, 80%, and 100% respectively. At the segment level, sensitivity, specificity, PPV, and NPV in the premature beat group were 92.55%, 98.21%, 88.51%, and 98.72% respectively, while those in the control group were 95.79%, 98.42%, 90.11%, and 99.28% respectively. Between the two groups, specificity, sensitivity, PPV, NPV was no significant difference. In the premature beat group, 10 coronary artery segments had false positive(FP) results and 7 segments had false negative(FN) results. In the control group, 9 coronary artery segments had false positive results and 4 segments had false negative results (Table 3).

**Table 3:** Coronary artery stenosis diagnosis accuracy in the premature beat group and the control group.

	Premature beat group		Control group	
	Patient level (50)	Segment level (635)	Patient level (50)	Segment level (650)
TP	15	87	10	91
TN	35	548	40	559
FP	2	10	2	9
FN	0	7	0	4
sensitivity	100%	92.55%	100%	95.79%
specificity	94.95%	98.21%	95.24%	98.42%
PPV	86.66%	88.51%	80%	90.11%
NPV	100%	98.72%	100%	99.28%

### Image Quality

M-field scanning coronary segment image scores of 4, 3, 2, and 1 for 50 cases in the premature beat group account for 602 (94.80%), 28 (4.41%), 5 (0.78%), and 0 cases respectively. Those same scores in the control group account for 615 (94.61%), 31 (4.76%), 4 (0.61%), and 0 cases respectively. The difference between the two groups had no statistical significance (P>0.05, Table 4). In both groups, scores of 3 and 2 were caused by higher heart rate and poor breath holding. The segments with scores of 1 were not evaluated.

**Table 4:** Coronary artery image quality scores.

	4	3	2	1
Premature beat group	602 (94.80%)	28 (4.41%)	5 (0.78%)	0
Control group	615 (94.61%)	31 (4.76%)	4(0.61%)	0

### Heart Rate, Dose, and Scanning Field Selection

The mean heart rate (77.20±12.07 bpm) and radiation dose (14.62±1.37 mSv) in the premature beat group were higher than the mean heart rate (58.72±4.73 bpm) and radiation dose (3.08±2.35 mSv) in the control group (P<0.05, Table 5). Heart rate and radiation dose in the premature beat group were significantly correlated at a level of confidence (both sides) of 0.01 (Pearson correlation coefficient  = 0.699, P<0.001). Heart rate and radiation dose in the control group, however, displayed no such no correlation (Pearson correlation coefficient  = 0.048, P>0.05). In the premature beat group, based on heart size, 10 subjects should have received S-field scanning, but the scanning field was artificially changed to M-field. These patients were compared to 10 patients with S-field scanning in terms of radiation dose and image quality. The results of this comparative analysis showed that the radiation dose in S-field scanning is significantly higher than that in M-field scanning, while there was no difference in image quality (Table 6).

**Table 5:** Comparison of statistical results of variable parameters in the premature beat group and the control group.

	N	BMI (kg/m^2^)	Age	heart rate (bpm)	score
Premature beat group	50	25.47±2.89	61.04±10.71	77.20±12.07	3.93±0.25
Control group	50	25.54±2.62	57.80±10.40	58.72±4.73	3.94±0.30
*P* value		0.498	0.837	0.000	0.205

*P*<0.05 indicates a statistically significance difference.

**Table 6:** Comparison of results from M-field scanning and S-field scanning in the premature beat group.

	N	heart rate(bpm)	BMI(kg/m^2^)	dose*(mSv)	score
M-field	10	82.10±16.05	25.41±2.91	15.10±1.12	3.93±0.36
S-field	10	82.30±14.14	25.26±2.67	34.55±7.12	3.95±0.27
*P* value		0.712	0.801	0.000	0.404

* Dose conversion coefficient k = 0.014 mSv/mGy×cm [18], [19], *P*<0.05 indicates a statistically significance difference.

## Discussion

### CTCA Diagnostic Accuracy

**Figure 2:**
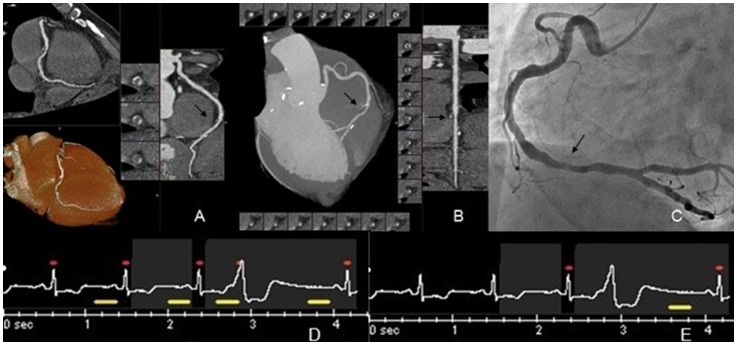
A male patient, 58-years-old, with an image quality score of 4. Figure A includes VR and CPR images, and Figure B includes an MIP image and a CPR straightened image used when measuring degree and range of luminal stenosis. The images show multiple soft plaques and calcified plaques at the right coronary and moderate stenosis at distal lumen (indicated by the superimposed arrow). Figure C is an example of coronary angiography, which demonstrates that it is in accordance with the results of CTCA. Figure D is an example of the ECG taken when data was acquired. Figure E show the ECG-edit later.

It can be seen from the results that sensitivity, specificity, PPV, and NPV in both groups are high, and there is no significant differences between the two groups. CTCA can accurately diagnosis CAD, and is particularly effective in excluding CAD. With the increasing strength of post-processing technology, we can observe the position, degree, and range of lesions from all angles and directions. After straightening the coronary artery at the lesion site, we can more accurately observe the degree and range of stenosis. We can also more accurately guide the selection of stent positioning in clinical settings (Figure 2). Before stent positioning, CAG is not sensitive enough to detect mild stenosis caused by small plaques. Because of this limitation, if the range of stent positioning is not sufficient, incidence of restenosis will be significantly higher. Therefore, multi-detector spiral CT has been the first choice for clinical diagnosis and treatment of CAD.

Currently, the main factor affecting diagnostic accuracy is the artifacts caused by heart rate and breathing. Additionally, the lumen covering caused by high-density and large-area calcified plaques and metal stents also increases the severity of artifacts, and as a consequence impacts the ability to correctly access the degree of stenosis. Among those patients investigated by this study who had premature beats, 10 coronary segments gave false positive results. Of those, two were at the left anterior descending (LAD), three were at the left circumflex (LCX), and two were at the right coronary artery (RCA). In these cases calcified plaques and a metal stent leading to lumen caused an over-estimation in the true degree of stenosis. The remaining three false positives (two at the LCX and one at the RCA) were the product of motion artifacts that affected assessment accuracy. Seven false negative results were due to poor image quality and calcified plaques. In the control group, nine coronary segments gave false positive results. Of those, five were at the LAD and four were at the LCX. These false positives were caused by large areas of calcified plaque that resulted in an over-estimation of the true degree of stenosis. In the four false negative results, two were at the LCX and two were at the RCA. These were the result of artifacts being mistaken as plaques.

### Image Quality

**Figure 3:**
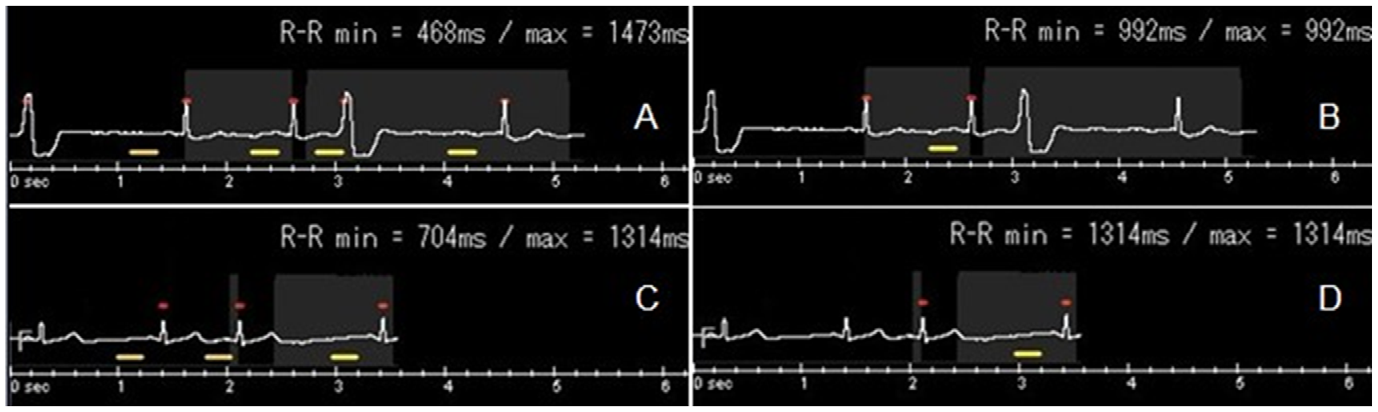
Two patients with ventricular premature beats. Figure A shows that heart rate is 71 bpm, 2-beat scanning is adopted, and acquisition is at 30–80% of R-R interval. A premature beat is encountered at the second beat during scanning. The scanning does not stop, and continuously scans the next R-R interval. Figure C shows the heart rate to be 55 bpm, 1-beat scanning is adopted, and acquisition at is 70–80% of R-R interval. Scanning is stopped immediately when a premature beat in encountered, and the scan is resumed at the next normal cardiac cycle. Figures B and D show the ECG-edit of A and C respectively.

Along with the rapid development of multi-detector spiral CT, 320-detector (640-slice) spiral CT can be seen as the highest level of multi-detector CT. A single scan can cover the whole heart, avoiding the stair-step artifacts that are associated with in 64-detector CT. In addition to the reconstruction of more accurate 3D images [12] and CTA images with higher spatial resolution [13], it also allows for some high-tech applications. For example, arrhythmia scanning software can intelligently identify premature beats, and when checking subjects with premature beats, the software can maximize image quality while reducing radiation dose. If ventricular premature beats are encountered when scanning the first R-R interval, the equipment will automatically stop scanning and collect data at the next period phase. If ventricular premature beats are encountered when scanning R-R intervals after the second interval, the equipment will continue scanning until the end of the next R-R interval (Figure 3). It is very important to ventricular premature beats, and they are abnormal in only one cardiac cycle, so by omitting the R-R interval with premature beat and scanning normal R-R intervals, the necessary period phase intervals can be collected. This is especially important for patients with heart rates below 65 bpm. This is because when a person’s heart rate is below 65 bpm, the equipment will collect a 70%–80% interval of a cardiac cycle. If scanning cannot be stopped during this period, the interval collected would not truly represent 70%–80% of a cardiac cycle. As a consequence, high-quality images cannot be reconstructed. However, 320-slice CT can automatically collect the data from 30%–80% of the next cardiac cycle, thereby avoiding scanning failure and ensuring high image quality.

### Radiation Dose

In order to increase time resolution, when scanning the equipment’s beat value should correspond to patient heart rate within a certain range [14], [15]. The equipment’s default ranges are as follows: <65 bpm, 1 beat; 65–80 bpm, 2 beats; 81–117 bpm, 3 beats;118–155 bpm, 4 beats; and >156 bpm, 5 beats. Therefore, the higher the beat value, the longer the acquisition time and higher the dose of radiation. Pearson correlation coefficient analysis shows that radiation dose and heart rate in the control group had no statistically significant correlation (correlation coefficient = 0.048). This is likely because heart rate range in the control group (45 to 64 bpm) was narrow, and scanning was completed in a single heart beat (half scan in 1 beat). Only differences in individual body mass index lead to differences in tube voltage and tube current. Therefore differences in radiation dose between individuals in this group has no statistical significance. However, in the premature beat group, heart rate range was larger (55∼106 bpm), so radiation dose and heart rate showed a moderate correlation (correlation coefficient = 0.699).

The radiation dose in the premature beat group was significantly higher than that in the control group because when an individual’s heart rate is higher better images can usually be reconstructed at the end of systolic period [16], [17]. For this reason, acquisition was performed from 30% to 80% time phase in R-R intervals in the premature group. Conversely, in the control group only 70%–80% time phase was collected. As a result, the radiation dose (14.62±1.37 mSv) in the premature beat group was about five times higher than the dose (3.08±2.35 mSv) in the control group.

The size of scanning field is adjusted in accordance with the size of the patient’s heart. As a rule, the smaller the scanning field, the higher the spatial resolution of images. When S-field is used in scanning, the equipment’s default reconstruction mode is for brain, and higher spatial resolution is required. When M-field is used in scanning, the default reconstruction mode is for body, and the required spatial resolution reduces, so the radiation dose correspondingly reduces as well. Usually heart size precludes the use of L-field scanning. In the premature beat group, based on heart size, 10 subjects should have received S-field scanning, but the scanning field was artificially changed to M-field. These 10 subjects were compared against 10 subjects who underwent S-field scanning. This study found that there was no statistical difference in image quality or other variables between these two groups. Only the radiation dose in S-field scanning was significantly higher than that in M-field scanning. Considering the health of the patients, the control group in this study all underwent M-field scanning. The artificial change of scanning field in this group is not compared and analyzed. However, in theory, M-field scanning can preserve image quality while significantly reducing radiation dose.

### Conclusions

In summary, by taking the advantage of a 320-detector CT scanner’s ability to compensate for premature beats, and then manually adjusting scanning field size to the M-field range, high diagnostic accuracy can be maintained while limiting radiation dose.
